# Retrospective cohort study of type 2 diabetes effects on chronic wound healing in dermatology clinics

**DOI:** 10.6026/973206300220853

**Published:** 2026-02-28

**Authors:** M.R. Madan Karthik Raj, Sailesh I.S Kumar, Shanmukha Koppolu, Vijo Wilson

**Affiliations:** 1 Department of General Surgery, Vinayaka Mission's Kirupanandha Variyar Medical College and Hospital, Salem, Tamil Nadu, India; 2 Department of General Medicine, Madras Medical College, Tamil Nadu, India; 3 Department of Trauma and Orthopaedics, Barts NHS Trust, London, England, United Kingdom; 4 Department of General Medicine, Wexham Park Hospital, Frimley NHS Trust, Foundation Trust, Berkshire, United Kingdom

**Keywords:** Type 2 diabetes mellitus, chronic wound healing, dermatology clinics, retrospective cohort, wound infection, glycemic control, multidisciplinary care

## Abstract

Chronic Wounds are one of the most prevalent types of Dermatoses, which are the result of impaired Healing and Complications. In addition, Type 2 Diabetes Mellitus is one of the main causes of impaired Healing (due to the development of Complications and other factors). Therefore, it is of interest to evaluate the way T2DM influences Healing, Recurrence and Clinical Outcomes (in Dermatology Clinics) over a period of five years. A retrospective review of medical records (of chronic wound patients) identified differences between the T2DM and the Non-Diabetic Groups. The T2DM patients demonstrated a significantly longer median Healing Time than the Non- Diabetic Patients, with increased Infection Rates and an increased need for Advanced Wound Care. The rate of Recurrence and Amputation were also higher than in the Non-Diabetic Group. A Multivariate Analysis indicated that T2DM, Baseline Wound Infection and Wound Size >10 cm^2^ were significant predictors of Delayed Healing for T2DM Patients. Thus, these data demonstrate that T2DM places a significant burden on Wound Outcomes in a Dermatology Practice. Early identification of risk factors, multidisciplinary management and optimised glycaemic control are crucial to improving Healing and reducing complications associated with Chronic Wounds and T2DM.

## Background:

Chronic wounds are defined as the failure to progress through the phases of healing in a timely manner. Chronic wounds comprise an overwhelming amount of the global disease burden and contribute substantially to prolonged morbidity, healthcare expenditures [1]. In the clinic setting, Dermatology clinics manage a large number of chronic wound patients in both outpatient and inpatient venues [2]. Chronic Wounds can be attributed to a variety of causes, including diabetic foot ulcers, venous leg ulcers, arterial ulcers, pressure ulcers and traumatic/post-operative wounds [3]. Regardless of the aetiology, all chronic wounds show common characteristics of impaired tissue repair and prolonged inflammation [4]. Type 2 Diabetes Mellitus (T2DM) is one of the most significant overarching conditions associated with delayed wound healing [5]. Due to the increasing age demographic, urbanisation and changing lifestyles, the incidence of T2DM globally continues to rise. Hyperglycemia interferes with many biological mechanisms critical to tissue repair; For example, advanced glycation end products (AGEs) disrupt the structural integrity of the extracellular matrix (ECM) and affect the cellular signalling pathways [6]. Microvascular / macrovascular disease alters the delivery of oxygen and nutrients to tissues. Diabetic patients with neuropathy often sustain repeated trauma due to their inability to perceive injuries [7]. Impairment of immune function adds to the already impaired ability of neutrophils to migrate to the site of injury, to phagocytose invading pathogens and to produce the appropriate mediators to promote growth and healing. These combined mechanisms create an environment for sustained inflammatory reaction that ultimately enhances the formation of granulation, angiogenesis and epithelialisation [8]. In many cases chronically healed diabetic wounds have a delayed time to heal, increased risk for infection, higher recurrence and amputation rates. Management of diabetic wounds frequently requires advanced therapeutic options such as surgical debridement; negative pressure wound therapy and the use of skin substitutes which are bioengineered. Comorbidities related to chronic wound development such as chronic kidney disease, peripheral arterial disease and obesity add to the complexity of care [9]. A multidisciplinary approach involving dermatology, endocrinology and vascular specialists will be needed to effectively care for people with chronic wounds. The available evidence regarding the care of diabetic wounds is primarily obtained from the surgical and vascular clinic populations with minimal data concerning therapeutic options available through dermatology clinics [10]. Dermatology services provide early diagnosis, specialized wound assessment and access to advanced topical and procedural treatment options in the acute care setting. In summary, accurately interpreting the actual clinical pathways for managing diabetic wounds can only be achieved through the application of real-world clinical experience from dermatology practice settings [11]. Therefore, it is of interest to evaluate the effect of T2DM on healing outcomes of chronic wounds managed in dermatology clinics. The primary focus is healing time, recurrence, complications and need for advanced interventions.

## Materials and Methods:

To study the impact of type 2 diabetes mellitus (T2DM) on the outcome of healing of chronic wounds, retrospective cohort studies were carried out in the dermatology outpatients of a tertiary care facility. The study consisted of reviewing patient files from January 2019 to December 2023. Adult patients aged 18 years and older with chronic wounds of more than six weeks were taken into consideration for enrollment. Chronic wounds were diabetic foot ulcers, venous leg ulcers, arterial ulcers, pressure ulcers and postoperative or traumatic non-healing wounds. Inclusion criteria required complete medical records documenting wound characteristics, comorbidities, treatment interventions and follow-up until healing or last clinical review, with a minimum follow-up of 12 weeks. Patients with type 1 diabetes, gestational diabetes, malignant ulcers and wounds secondary to dermatological malignancies, immunocompromised states or incomplete documentation were excluded. Data were extracted from electronic health records and patient charts using a standardized proforma. Information collected included demographic details (age, sex, occupation), medical history (duration and control of T2DM, comorbidities such as hypertension, peripheral arterial disease, chronic kidney disease and obesity), wound characteristics (etiology, anatomical location, size, depth, infection status and duration prior to presentation), clinical interventions (type of dressing, systemic antibiotic use, surgical procedures such as debridement or grafting and adjunctive therapies such as negative pressure wound therapy or bioengineered skin substitutes) and outcome measures (time to complete epithelialization in weeks, recurrence within one year, wound- related complications including infection, hospitalization and limb amputation). The primary outcome was median time to complete wound healing, defined as full epithelialization without drainage. Secondary outcomes included recurrence rates, frequency of secondary infections, need for advanced wound care interventions and amputation rates. Continuous variables were expressed as mean ± standard deviation or median with interquartile range, depending on distribution and compared using t-tests or Mann-Whitney U tests. Categorical variables were summarized as frequencies and percentages, with comparisons using Chi-square or Fisher's exact tests. Kaplan- Meier survival analysis with log-rank testing was used to compare healing times between groups. A p-value of <0.05 was considered statistically significant.

## Results:

A study of 482 patients' records included 238 patients with type 2 diabetes mellitus (T2DM) and 244 patients without T2DM. While diabetic patients were generally older and had longer periods between injury and treatment than non-diabetic patients, there were significant differences between the two groups in the types of wounds that were experienced. Most diabetic patients developed diabetic foot ulcers, whereas most non-diabetic patients had either venous leg ulcers, traumatic wounds or postoperative wounds. Although both groups developed wounds on their lower extremities, diabetic patients developed more wounds on their plantar surfaces and toes. In addition, there was a significantly larger size of wound and a higher percentage of infections at the time of treatment in diabetic patients than in non-diabetic patients. Results of microbiological testing indicated that the organisms most often isolated from patients with diabetic foot ulcers were *Staphylococcus aureus* and *Pseudomonas aeruginosa* and that the incidence of polymicrobial infections was greater among diabetic patients. More advanced treatment procedures such as surgical debridement, negative pressure wound therapy, skin grafting and bioengineered skin substitutes were performed more frequently on diabetic patients than on non-diabetic patients. Diabetic patients also had longer delays in achieving wound healing than non-diabetic patients, had higher rates of recurrence of their wounds and were at greater risk of lower limb amputation than non-diabetic patients. The frequency and length of stay for hospital admissions and readmissions for diabetic patients were all higher than for non-diabetic patients. Results from the multivariate analysis indicated that T2DM, the presence of wound infection at the time of treatment and the size of the wound (greater than 10 cm^2^) were independent predictors of delayed healing time. Table 1 demonstrates that patients with T2DM were significantly older, presented later in the course of their wound and had a higher prevalence of comorbidities such as hypertension and peripheral arterial disease, while sex distribution was similar between groups. Table 2 shows that diabetic foot ulcers were the most common wound type in the T2DM cohort, whereas venous leg ulcers and traumatic or postoperative wounds were more prevalent among non-diabetic patients. Table 3 demonstrates that lower limb wounds predominated in both groups, particularly in T2DM patients, who also had higher rates of plantar surface and toe involvement compared to non-diabetics. Table 4 demonstrates that patients with T2DM had significantly larger wounds at presentation and a higher proportion of infected wounds compared to non-diabetic patients. Table 5 shows that *Staphylococcus aureus* and *Pseudomonas aeruginosa* were the most frequently isolated organisms in both groups, with a higher prevalence of polymicrobial infections in the T2DM cohort. Table 6 demonstrates that diabetic patients more frequently required advanced interventions such as negative pressure wound therapy, surgical debridement and skin grafting compared to non-diabetic patients. Table 7 demonstrates significantly delayed healing in T2DM patients, along with higher recurrence rates and increased risk of lower limb amputation compared to the non-diabetic group. Table 8 shows that T2DM patients had a higher rate of wound-related hospital admissions, longer inpatient stays and more frequent readmissions compared to non-diabetic patients. Table 9 demonstrates that wound-related complications such as cellulitis, osteomyelitis and sepsis were more frequent among T2DM patients. Table 10 shows that T2DM, baseline wound infection and wound size greater than 10 cm^2^ were independent predictors of delayed wound healing after adjusting for confounding variables.

## Discussion:

This retrospective cohort study examined how T2DM affects chronic wound healing at a dermatology clinic. The study found that chronic wound healing in patients with T2DM is delayed and has higher rates of complications and a greater need for outpatient care than those without T2DM. Therefore, the study results confirm the previously described effect of T2DM on tissue costs of repair within a real-world dermatology practice [12]. Additionally, patients with T2DM are older and tend to seek medical attention later in their disease progression. Patients with T2DM are also more likely to have hypertension (high blood pressure) and peripheral artery disease (PAD). The combination of vascular declines due to age and metabolic derangements may also be factors in delayed wound healing. Patients' late presentation is likely due to the loss of protective sensation from neuropathy and/or delayed recognition of symptoms [13]. Additionally, the aetiology of wounds in patients with DM and those without DM differs; diabetic foot ulcers were the most common type of wound among patients with T2DM, while leg ulcers due to venous insufficiency and wound-related injuries (or post-operative) were the most common types among non-DM patients. These differences reflect the different pathophysiological processes and risk profiles for each group of patients, thus necessitating different preventative strategies based upon the wound type's present [14]. The anatomical distribution for both groups of patients demonstrated the area of greatest risk for developing wounds was the diabetic foot, with plantar and toe wounds occurring much more frequently among T2DM patients than among non-diabetic patients, likely occurring due to increased frequency of exposure to repetitive pressure and shear stress. Neuropathy and impaired perfusion are the reasons for increased susceptibility to ulcertion and prolonged healing time and therefore protective footwear and off-loading techniques are critical in preventing such wounds [15]. The baseline wound characteristics for both groups of patients were significantly different; patients with T2DM started with larger ulcers and had greater infection rates than non-T2DM patients. The chronic hyperglycaemia of DM can impair the function of white blood cells and decrease their ability to kill bacteria. In immune compromised individuals, the microorganisms are likely to colonize abundantly. This ultimately results in persistent inflammation and an environment incapable of producing granulation and epithelialization [16].

Microbiological cultures have shown that *Staphylococcus aureus* (*S. aureus*) and *Pseudomonas aeruginosa* (*P. aeruginosa*) were isolated more frequently than other organisms. Furthermore, diabetic wounds (type 2 diabetes mellitus; T2DM) have increased occurrence of polymicrobial infections (more than one type of infectious organism). Thus, the presence of a polymicrobial infection increases tissue destruction and the complexity of treatment while requiring continued use of antibiotics and repeating surgical debridement. Overall, the treatment patterns for diabetic wounds suggest that the extent of wound severity is considerably higher among patients with T2DM than non-diabetic patients. In particular, the advanced interventions of surgical debridement, negative pressure therapy and skin grafting were used with greater frequency among T2DM patients [17]. These types of interventions are most often reserved for complex, non-healing wounds; therefore, their increased use clearly indicates the burden that diabetic wounds pose on the clinical environment. Unfortunately, the rate of healing was substantially lower for diabetic wounds than that of the non-diabetic cohort. In addition, the median healing time was significantly longer for diabetic patients and there was a significantly higher recurrence of wounds in T2DM patients [18]. Furthermore, the risk of amputation was considerably higher when compared to those without diabetes. The above circumstances are primarily due to the complex interrelated nature of vascular insufficiency, neuropathy and infection susceptibility. The frequent recurrence of diabetic ulcers poses a specific concern to limb loss, since recurrent ulcers present an increased risk for amputation [19]. Furthermore, the hospitalization burden on diabetic patients is much greater in comparison to non-diabetic patients. Specifically, T2DM patients experienced a greater number of admissions, longer lengths of stay and higher rereadmission rates than their non-diabetic counterparts. This again demonstrates the increased utilization of healthcare and the subsequent economic impact on the healthcare system. Hence, to reduce the system wide impact of diabetic wounds, it is crucial to initiate preventative (preventative care) measures and the early treatment of these wounds [20]. Diabetic patients experienced more complications due to complications related to or caused by necrotizing fasciitis and the rates of cellulitis, osteomyelitis and septicemia were all significantly increased compared to non-diabetic patients. The presence of osteomyelitis is a major contributor to the need for prolonged treatment and/or amputations. Therefore, early control of infections, monitoring of patients with diabetes and close follow-up of all patients who present with suspected necrotizing fasciitis are critical components in the immediate management of high-risk patients [21]. Using multivariable regression analysis, T2DM, the presence of a baseline infection at the time of consultation and a wound greater than 10 centimetres (cm) squared in size were found to be independent risk factors for delayed healing. Collectively, these three factors demonstrate that systemic disease (T2DM) interacts with local wound characteristics to delay healing of the wound. Identifying these independent predictors would provide the basis for the future development of risk stratification tools. Therefore, it is prudent to prioritize aggressive early management of patients considered at high risk for delayed healing [22]. The present investigation provides a real-world perspective, providing clinic-specific evidence regarding the patient outcomes for chronic traumatic wounds among diabetic patients. In addition, the study quantified the differences among diabetic and non-diabetic patients regarding healing time, abnormal recurrence rates, hospitalization burden and risk of amputations. The identified independent predictors for a delay in healing were identified through the use of a multivariable regression analysis. Overall, these data provide support for a risk-stratified-multidisciplinary approach for the management of chronic traumatic wounds in the dermatology clinical setting. Limitations of this study include the fact that the study utilized a retrospective study design, which may result in an under-reporting of patient care due to the documentation bias. Data used for this research were exclusively obtained from a single center (dermatology clinic). Additionally, some of the variables, including patient socioeconomic status and treatment adherence, were not available for examination. Microbiological testing was conducted only when clinically indicated. Despite these limitations, the study has demonstrated real-world clinical applicability of data obtained from dermatology practice.

## Conclusion:

Type 2 diabetes mellitus significantly delays healing and increases complications in chronic wounds managed in dermatology clinics. Early risk stratification, strict infection control and multidisciplinary care are essential to improve healing outcomes and reduce long-term morbidity.

## Figures and Tables

**Table 1:** Baseline demographic and clinical characteristics of study participants

**Parameter**	**T2DM (n = 238)**	**Non-diabetic (n = 244)**	**p-value**
Mean age (years) ± SD	59.3 ± 11.7	56.4 ± 13.0	0.012
Male sex, n (%)	134 (56.3)	127 (52.0)	0.349
Duration of wound before presentation (weeks), median (IQR)	8.2 (6.5-11.4)	6.1 (4.8-8.7)	<0.001
Hypertension, n (%)	149 (62.6)	95 (38.9)	<0.001
Peripheral arterial disease, n (%)	66 (27.7)	35 (14.3)	<0.001

**Table 2:** Distribution of wound etiology between T2DM and non-diabetic groups

**Wound type**	**T2DM (n = 238)**	**Non-diabetic (n = 244)**	**p-value**
Diabetic foot ulcer	111 (46.6)	-	-
Venous leg ulcer	51 (21.4)	96 (39.3)	<0.001
Arterial ulcer	28 (11.8)	21 (8.6)	0.281
Pressure ulcer	26 (10.9)	18 (7.4)	0.206
Traumatic/postoperative non-healing wound	22 (9.2)	109 (44.7)	<0.001

**Table 3:** Anatomical location of chronic wounds

**Location**	**T2DM (n = 238)**	**Non-diabetic (n = 244)**	**p-value**
Plantar foot	88 (37.0)	21 (8.6)	<0.001
Toes	47 (19.7)	9 (3.7)	<0.001
Ankle/Lower leg	56 (23.5)	112 (45.9)	<0.001
Upper limb	12 (5.0)	18 (7.4)	0.297
Trunk	35 (14.7)	84 (34.4)	<0.001

**Table 4:** Microbiological profile of infected wounds

**Parameter**	**T2DM (n = 238)**	**Non-diabetic (n = 244)**	**p-value**
Mean wound size (cm^2^)± SD	14.8± 7.5	10.9± 6.2	<0.001
Infected wounds at presentation, n (%)	138 (58.0)	89 (36.5)	<0.001

**Table 5:** Wound healing interventions used

**Isolated organism**	**T2DM (n = 138 infections)**	**Non-diabetic (n = 89 infections)**	**p-value**
*Staphylococcus aureus*	54 (39.1)	26 (29.2)	0.134
*Pseudomonas aeruginosa*	37 (26.8)	21 (23.6)	0.613
*Escherichia coli*	21 (15.2)	11 (12.4)	0.564
Polymicrobial	44 (31.9)	14 (15.7)	0.009

**Table 6:** Wound healing outcomes

**Intervention**	**T2DM (n = 238)**	**Non-diabetic (n = 244)**	**p-value**
Standard dressings only	78 (32.8)	156 (63.9)	<0.001
Surgical debridement	142 (59.7)	83 (34.0)	<0.001
Skin grafting	54 (22.7)	19 (7.8)	<0.001
Negative pressure wound therapy	46 (19.3)	18 (7.4)	<0.001
Bioengineered skin substitutes	14 (5.9)	5 (2.0)	0.027

**Table 7:** Hospitalization patterns

**Outcome**	**T2DM (n = 238)**	**Non-diabetic (n = 244)**	**p-value**
Median healing time (weeks), IQR	14 (11-18)	10 (8-13)	<0.001
Recurrence within one year, n (%)	51 (21.4)	29 (11.9)	0.004
Lower limb amputation, n (%)	18 (7.6)	5 (2.0)	0.003

**Table 8:** Hospitalization patterns

**Parameter**	**T2DM (n = 238)**	**Non-diabetic (n = 244)**	**p-value**
Any wound-related hospitalization, n (%)	112 (47.1)	69 (28.3)	<0.001
Mean length of stay (days) ± SD	9.6 ± 4.2	6.3 ± 3.1	<0.001
Readmission within 6 months, n (%)	34 (14.3)	12 (4.9)	0.001

**Table 9:** Complication profile during follow-up

**Complication**	**T2DM (n = 238)**	**Non-diabetic (n = 244)**	**p-value**
Cellulitis	67 (28.2)	34 (13.9)	<0.001
Osteomyelitis	29 (12.2)	8 (3.3)	<0.001
Sepsis	12 (5.0)	4 (1.6)	0.047

**Table 10:** Independent predictors of delayed wound healing (Cox proportional hazards model)

**Variable**	**HR**	**95% CI**	**p-value**
T2DM	0.64	0.53-0.78	<0.001
Baseline wound infection	0.71	0.59-0.85	0.001
Wound size >10 cm^2^	0.68	0.55-0.82	<0.001
